# The Artificial Intelligence in Digital Pathology and Digital Radiology: Where Are We?

**DOI:** 10.3390/healthcare9010030

**Published:** 2020-12-31

**Authors:** Daniele Giansanti

**Affiliations:** Centre Tisp, Istituto Superiore di Sanità, Via Regina Elena 299, 00161 Roma, Italy; daniele.giansanti@iss.it; Tel.: +39-06-4990-2701

## 1. The Digital Radiology and Digital Pathology

Thanks to the incredible changes promoted by Information and Communication Technology (ICT) conveyed today by *electronic-health* (*eHealth*) and *mobile-health* (*mHealth*), many new applications of both organ and cellular diagnostics are now possible. Today, in the era of digitalization, we prefer to speak specifically about the prospects of *digital radiology* and *digital pathology*.

The first one includes the use of diagnostic imaging tools for organs and/or body functions based on systems compatible with *Digital Imaging and COmmunications in Medicine* (DICOM), or as it is commonly said, *DICOM-compliant*. In particular, *digital radiology* (DR) includes not only those instruments whose image formation processes are based on the interaction fields that use ionizing radiation, but also instruments of different types such as, for example, those instruments based on ultrasound (ultrasound) or magnetic fields [1,2] (nuclear magnetic resonance).

The second one, *digital pathology* (DP) includes the use of digital processes related to instrumentation for cellular diagnostics, mainly (but not only) in two forms: histological and cytological [3,4]. We can also speak of *digital histology* and *digital cytology* as the two major components of the DP, however, the DP also includes other processes for digitizing information in a biomedical laboratory such as by way of non-exhaustive example such as those relating to the integration of cytometric reports.

## 2. General Actual Developments of *Digital Radiology*, *Digital Pathology*, and the Artificial Intelligence

Of course, a detailed analysis of the perspectives of the DR and the DP would deserve two separate treatments. However, in light of the objective of this contribution, brief considerations of a fundamental nature are reported. In recent years, the DR has opened up both to new forms of construction and/or reconstruction applications of artificial reality and artificial intelligence (also related).

As far as artificial reality is concerned, we are seeing more and more applications of augmented and/or virtual reality. For example, in [5,6], they are some available ones that use the DICOM file from computed tomography) and/or magnetic resonance imaging to return to the surgeon on support augmented reality viewers during an operation (as for a fighter pilot), for example, the exact placement of blood vessels or a nerve. Virtual reality applications in the so-called virtual colonoscopy or in all those endo-cavitary diagnostic applications, where it is possible to create a real virtual journey (thanks to the processing of *voxels* starting from the file saved in DICOM), are now routine. This possibility of processing *voxels* to create environments of artificial reality also finds wide application today in the two sectors of three-dimensional reconstruction (3D) and simulation and training in surgery. In fact, by processing the *voxels*, it is possible to transform the DICOM file into a file of the standard format for the 3D printing called stereolithography (STL) and to print models of organs and tissues (e.g., bones). For example, it is now routine to print skeletal parts to design replacement bioengineering parts. An example of such applications can be found in the treatment of pelvic fractures caused by osteoporosis or bone cancer: the 3D model of the pelvis is printed and then bioengineering grafts are designed with biomaterials on the same model. As for simulation and training in surgery, it is now possible to use technologies for augmented and virtual reality combined with systems for restoring the perception of force, called force-feedback. Therefore, the system will give a perception of force-feedback associated, for example, with the liver to the surgeon who trains in liver surgery. Furthermore, *Artificial Intelligence* (AI) is bursting into the world of the DR and is affecting many scholars both in the world of technologies and bioethics. These scholars are interested both in the potential of feature recognition, automatic recognition, and quality control, but also in the limitations and related problems and the new applications in the fields described above and the related research activities [7,8,9]. Surely, the evolutions of digitalization processes in the world of the DP [3,4] are taking place at a slower rate than in the world of DR, especially because the DP has not adapted to the DICOM standard (DICOM WSI in this case) with the same readiness as the DR. However, it has been shown how digitization processes in this area [4,5,6] can favor the decision processes and the training, which uses a very strong component of *mHealth* in environments where training is based on smartphone viewers to which the image is sent from a centralized digital microscope. Furthermore, artificial reality can certainly be of support by providing tools to navigate around cellular and/or tissue elements. Furthermore, the AI, as discussed for the DR, is breaking into the world of the DP, and also in this case affecting many scholars both in the world of innovative technologies [10] and bioethics.

## 3. What Future Awaits Us for Artificial Intelligence in Digital Radiology and Digital Pathology?

Recent advances in AI have led to the conclusion that artificial intelligence could be very useful in DR very soon in routine applications [7]. Researchers have developed *deep learning neural networks* that can identify pathologies in radiological images such as bone fractures and potentially cancerous lesions [8,9]. The *deep learning* is advancing rapidly, and is a much better technology than previous approaches to medical imaging, however, the best systems currently live up to human performance and are used only in research environments. Radiological practice would certainly benefit from systems capable of rapidly reading and interpreting multiple images because the number of images has increased much faster in the last decade than the number of radiologists. It is evident that in DR, the amount of work is very high, so every solution able to reduce it could be welcome, decreasing the costs and improving the process. Like other AI systems, systems used in radiology perform individual tasks and are trained and used for specific image recognition tasks. However, thousands of activities are needed to fully identify all potential outcomes in medical images, and only some of these can be performed by the AI today. In addition, image interpretation work includes only a few of the tasks performed by radiologists. They also consult with other doctors for diagnosis and treatment, treat diseases, perform image-guided medical interventions, tailor the exam on the patient’s needs, discuss outcomes, activities, and procedures with patients and many other activities. *Therefore, in DR it is important to understand what the role of AI is and the help it can provide us.* Scientists in DP benefit from combining histopathological data obtained, analyzed, and shared with other sources of clinical data such as that obtained from *omics, historical clinical data*, and *demographic data*. However, it is difficult to integrate the data collected in different formats that do not combine in a useful way [3,4]. For example, medical records are mostly kept in an unstructured text format. AI is helping to integrate information from these multiple sources [5,6,10]. Natural language processing, a branch of AI, is being used, for example, to extract pertinent details from written notes from the entire slide representation. Methodologies of AI are being developed to help integrate data from all sources, not just imaging. In addition, AI is being used to help decrease the errors that are made in diagnostic pathology. Before the birth of DP, the diagnosis from tissue samples always rested on the competence of medical professionals. AI can try to reduce the error rate in the diagnostic processes.

## 4. What Are the Aspects to Be Explored and to Deepen?

It is clear that AI, albeit with a different speed, is making a major breakthrough in both DR and DP.

In both sectors:

- The development involves both the world of imaging diagnostics and the related ones described above.
- A large number of image databases and in any case data patterns in general are being developed on which researchers can build and test their models/architectures of AI.
- A new direction certainly concerns the fusion of the contemporary approach based on AI on organ and cellular/histological diagnostics and affect both the radiology and biomedical laboratory.
- *There has been a tremendous research activity and impulse during the Covid-19 pandemic [11]*.
- mHealth is emerging through the use of targeted Apps.

This incredible development involves many scholars both from the world of technologies and bioethics. These scholars are interested in both the potential in applications of AI in feature recognition, diagnostics, automatic recognition, integration in the new processes including the quality control, but also to the limits and the related problems. It is important to face and contribute to this, which has a broad spectrum ranging from the continuous innovation aspects including the recent ones correlated to the Covid-19 pandemic up to the problem of the last “yard” of the AI, depending on the acceptance of all actors from the health operators up to the patients.
